# Avatar and distance simulation as a learning tool – virtual simulation technology as a facilitator or barrier? A questionnaire-based study on behalf of Netzwerk Kindersimulation e.V.

**DOI:** 10.3389/fped.2022.853243

**Published:** 2022-10-26

**Authors:** Ruth M. Löllgen, Joana Berger-Estilita, Lisa A. Rössler, Lukas P. Mileder

**Affiliations:** ^1^ Pediatric Emergency Department, Astrid Lindgren Children’s Hospital, Karolinska University Hospital, Stockholm, Sweden; ^2^ Department of Women's and Children's Health, Karolinska Institute, Stockholm, Sweden; ^3^ Netzwerk Kindersimulation e.V., Tübingen, Germany; ^4^ Institute for Medical Education, University of Bern, Bern, Switzerland; ^5^ Centre for Health Technology and Services Research (CINTESIS), Faculty of Medicine of Porto, Porto, Portugal; ^6^ Division of Neonatology, Pediatric Intensive Care and Neuropediatrics, Department of Pediatrics, Comprehensive Center for Pediatrics, Medical University of Vienna, Vienna, Austria; ^7^ Division of Neonatology, Department of Pediatrics and Adolescent Medicine, Medical University of Graz, Graz, Austria

**Keywords:** simulation-based education, avatar simulation, distance simulation, virtual simulation, advantages, challenges, pediatric acute care

## Abstract

**Background:**

Virtual simulation modalities have been implemented widely since the onset of the severe acute respiratory syndrome coronavirus 2 pandemic restrictions in March 2020, as educators face persistent restrictions to face-to-face education of medical students and healthcare professionals.

There is paucity of published data regarding the benefits and barriers of distance and avatar simulation training modalities.

**Methods:**

Following a 2-day virtual pediatric simulation competition facilitated by Netzwerk Kindersimulation e.V., using remote human avatars and distance simulation, we conducted a multicenter survey to explore the advantages and challenges of avatar and distance simulation among participants. We used a modified Delphi approach to draft and develop the 32-item online questionnaire with 7-point Likert-like scales (7 being the highest rating).

**Results:**

Twenty participants answered our questionnaire. Respondents indicated both a high overall satisfaction (median of 5.0 [Q25–Q75: 4.0–6.0] ) for avatar and distance simulation 6.0 (5.0–6.0), respectively, as well as a high achieved psychological safety with both simulation types (5.0 [4.0–6.0] vs. 5.0 [4.0–6.0]). The most frequently reported profits of avatar and distance simulation included the elimination of travel distances, associated lower costs, less time spent attending the education activity, and effective communication and leadership training, especially with avatar simulation. Most often named challenges were technical problems, limited reception of non-verbal cues and a spatial distance from the team/educator.

**Discussion:**

Based on the results of this pilot study, avatar and distance simulation can be employed successfully and appear to be good supplements to face-to-face simulation. Other studies are warranted to further explore the effectiveness of various types of virtual simulation compared to conventional presential simulation. We suggest using avatar-based simulation for targeted communication and leadership skills training and the application of distance simulation to bring simulation experts virtually to remote places where educator resources are lacking.

## Background

Since March 2020, the ongoing pandemic caused by the severe acute respiratory syndrome coronavirus 2 has persistently put restrictions on simulation-based education (SBE) and training (1, 2). Simulation educators have turned to virtual, remote or hybrid training modalities to compensate for reduced exposure to simulated pediatric emergencies in places where traditional education had to be limited or cancelled.

The effectiveness of face-to-face (F2F) (also referred to as presential) SBE as a learning tool in healthcare has been widely described (3), with significant effects on knowledge, process skills, product skills, time skills and patient outcomes (4). However, only scant data are describing whether this powerful effect can also be achieved through virtual, remote or telesimulation modalities (5–8). There is a knowledge gap regarding the benefits and barriers of distance simulation (term used hereafter for reasons of clarity, but not excluding remote or telemedicine simulation modalities, unless the authors specifically refer to one of the other terms) and avatar simulation training modalities, with only limited published evidence (6, 9).

These terms are partly overlapping and interchangeable and have been defined as follows: Virtual simulation “… is where a real person operates simulated systems”, which may utilize avatars, i.e., virtual objects “used to represent a physical object (e.g., a human) in a virtual world” (10). Distance simulation refers to “implementing a simulation or training at a physical distance from the participant(s)”, while remote simulation is “… performed with either the facilitator, learners, or both in an offsite location separate from other members to complete educational or assessment activities” (10). For this purpose telesimulation may be used, which utilizes telecommunication and simulation resources to “provide education, training, and assessment to learners at an offsite location” (5).

More challenging aspects regarding avatar and virtual reality (VR) simulation, defined as “a computer-generated three-dimensional environment that gives an immersion effect” (10), include high purchase costs, physical side effects like visual asthenopia and motion sickness, and possible psychological side effects such as dissociation (11, 12).

Although distance simulation allowed simulation activities to continue while maintaining social distancing requirements, adaption to these circumstances differed between geographic regions with an Anglo-American/Anglo-Saxon and Indian vs. European preponderance regarding non-presential training modes (13). As the pandemic of the coronavirus disease 2019 (COVID-19) will continue to hinder F2F SBE and training, medical schools and teaching hospitals will have to continuously adapt and modify their educational activities to provide essential simulation training. While presential SBE will and shall not be replaced due to its proven benefits, understanding the benefits and barriers of distance simulation will help clinical educators and simulation trainers plan and deliver SBE during these challenging times.

### Aim of the study

We sought to explore potential advantages and obstacles with two different simulation training modalities (avatar and distance simulation) over conventional presential SBE among European simulation competition participants represented in our pediatric simulation network “Netzwerk Kindersimulation e.V.” (14). We hypothesized that there would be distinct but specific advantages with either modality compared to standard SBE that will be useful to know for educators and simulation trainers even for future SBE beyond the COVID-19 pandemic.

## Materials and methods

### Ethics

The Institutional Review Board at Karolinska University Hospital, Stockholm, Sweden (File number 2021 02983, June 29, 2021) waived the need for ethics approval. Participation in the survey was voluntary, and participation in the survey was considered “consent by participation”. We used ID numbers to code participants and requested no directly identifying data. We stored data in a secure repository accessible to the investigators only. As far as applicable, all procedures from this investigation followed the Helsinki Declaration (15). All researchers complied with the Data Protection Acts of their respective academic institutions.

### Study design and setting

We performed an international, prospective study using an online survey in the German language (Supplementary File 1: Participant evaluation virtual simulation competition survey in German). We translated the original survey to the English language for the purpose of publication (Supplementary File 2: Participant evaluation virtual simulation competition survey in English).

### Procedure

#### Simulation competition

We performed a 2-day virtual pediatric simulation competition (June 15–16, 2021) during the German Society for Neonatal and Pediatric Intensive Care Medicine (GNPI) 2021 annual conference (16), which was held virtually due to the third COVID-19 wave. In the virtual simulation competition, we enrolled four German-speaking teams from pediatrics, pediatric intensive care, and pediatric emergency medicine. Each team was composed of three specialists in training, one pediatric nurse and one specialist physician (*n* = 5).

**Figure 1:**
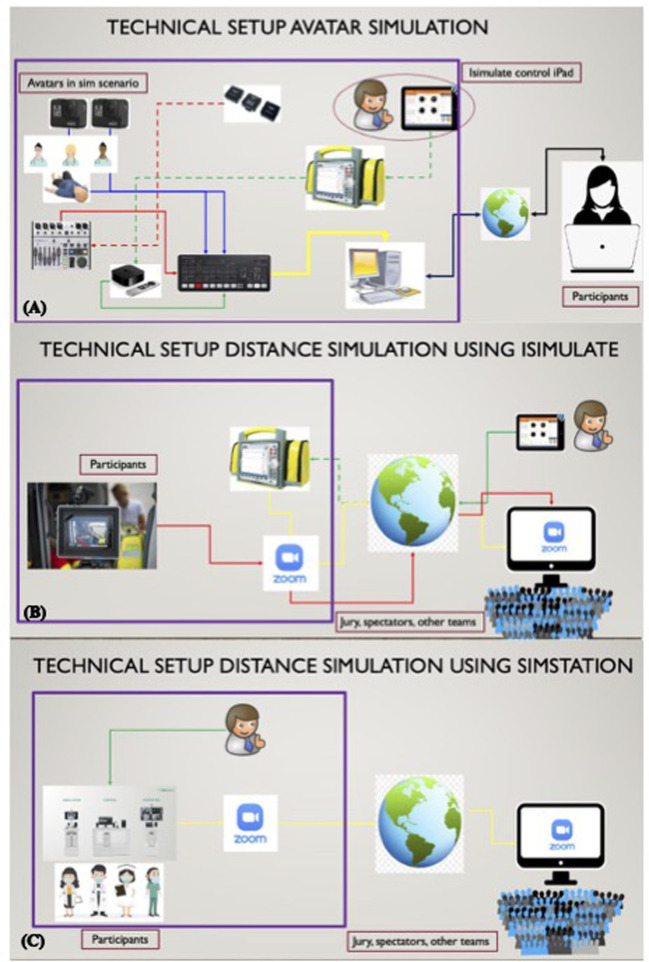
Concepts and technical setup of (**A**) avatar simulation and distance simulation using (**B**) iSimulate (iSimulate, Canberra, Australia and albany, New York, United States) or (**C**) SIMStation (©SIMStation, Vienna, Austria), respectively. Figures courtesy of Dr. Alberto Gyasi, Vienna, Austria. (**A**) On site: Control of the iSimulate monitor *via* the iSimulate control pad, which transmits the image of the monitor *via* radio to the Apple TV (Apple TV®, Apple Inc., United States) device. The Apple TV® device transmits the image to the video mixer [Atem mini (Blackmagic Design Pty. Ltd, Port Melbourne, Australia)] *via* HDMI. The two GoPro (©GoPro Inc., United States) cameras each transmit the action *via* HDMI to the video mixer. The Avatars transmit their sounds *via* wireless microphone to the wireless receiver. The wireless receiver feeds the sound to the video mixer *via* cable. The video mixer provides picture and sound to the participants *via* a virtual webcam over Zoom® (Zoom Video Communications, San Jose, California, United States). Participants side: Participants also use Zoom® to give instructions to the avatars *via* a speaker in the avatar room, allowing them to remotely control the avatars. (**B**) On-site: One iPad® (Apple Inc., United States) is used as an iSimulate patient monitor, another iPad® as a camera. Both must be connected to Zoom® to transmit the respective image. In the control center: The patient monitor can be controlled *via* an iPad®, which is connected to the monitor *via* internet. In addition, the two images from the remote simulation site are pinned (or brought into focus) on Zoom®. At a distance: That way the spectators and jury can follow the simulation. (**C**): On site: ©SIMStation (with inbuild cameras capturing the team and the monitor) needs to be connected to Zoom®. The patient monitor is controlled on site. At a distance: That way the spectators and jury can follow the simulation.

**For the qualification rounds,** we performed avatar simulations using the Zoom® platform (Zoom Video Communications, San Jose, California, United States) (Figure 1). An exclusive Zoom® meeting link was provided to the four participating teams, avatars, jurors (RML, Sweden, and one juror from each Switzerland and Germany) and the technician for the qualification round. The avatar simulations employed an avatar team of one nurse and two doctors as well as a technician physically located at St. Josef's Hospital in Vienna, Austria, performing an in-situ simulation scenario. The four participating teams were located remotely in Germany (*n* = 1, Münster) and Austria (Vienna *n* = 2, Eisenstadt *n* = 1). The avatars acted according to orders from the remotely participating teams, which were transmitted as voices into the room, in a simulation scenario with a pediatric emergency topic (supraventricular tachycardia).

**Figure 2:**
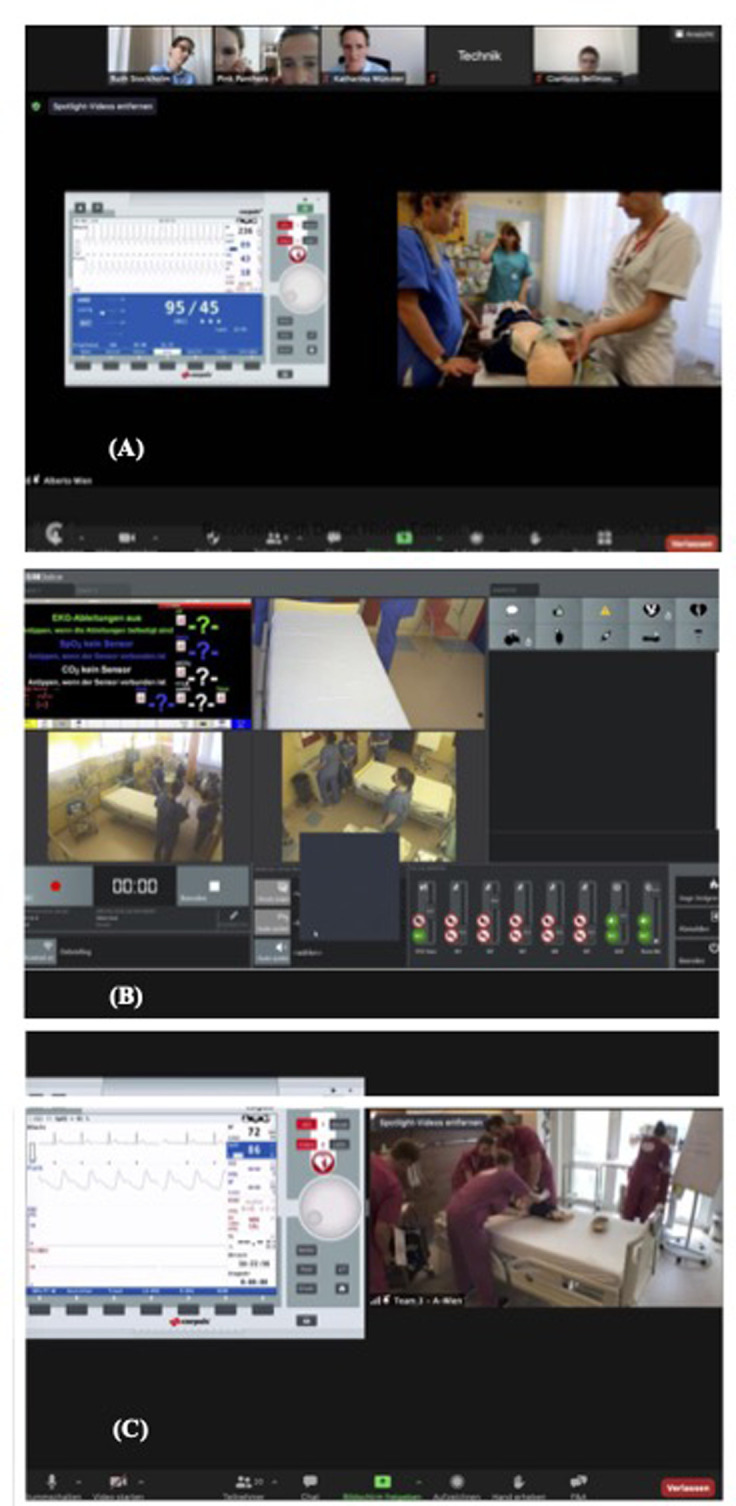
Participant and facilitator zoom®screen views. (**A**) Avatar simulation. Side-by-side format showing the operator's (technician's) shared screen of patient monitor vitals from a distance (located in Vienna), the avatars’ video of the simulated scenario and the gallery of participants, facilitators [jury members located in Stockholm (RML), Münster, Germany, and Bellinzona, Switzerland] and operator (technician), located in Vienna, Austria. Operator is in the same room and behind the camera. (**B**) Distance simulation using ©SIMStation. Side-by-side format showing the ©SIMStation's three camera views and local confederate operator's shared screen of patient monitor vitals transmitted *via* connection to Zoom®. (**C**) Distance simulation using iSimulate. Side-by-side format showing the operator's (technician's) shared screen of patient monitor vitals from a distance (Vienna) and the participants’ video of the simulated scenario transmitted by a camera connected to Zoom®.

**During the finals**, distance simulation was featured (Figure 2). We again used the Zoom® platform (Zoom Video Communications, San Jose, California, United States), and exclusive links were sent to the four participating teams, jurors, the technician for the final round and 12 registered passive spectators. All teams were challenged by two pediatric emergency simulation scenarios (drowning case for teams 1 and 2, status epilepticus case for teams 3 and 4). In this distance simulation, each team performed at their home institution, whether at the local simulation center (Münster) or *in situ* at the home hospitals (Comprehensive Center for Pediatrics, Medical University of Vienna; St. Josef's Hospital, Vienna; Hospital of the Brothers of Saint John of God, Eisenstadt) as per participant choice. For the distance simulation, the team's senior doctor acted as a confederate [defined as “an individual(s) who, during the clinical scenario, provides assistance locating and troubleshooting equipment” (10)]. The confederates received and accessed the scenario the day before the competition to (i) prepare the simulation manikin and training setting and (ii) operate the scenario for the competition according to the detailed script.

The virtual jury and organizing committee took part in the Zoom® competition to ultimately choose the winner by assessing medical and teamwork aspects. A specific 60-point evaluation tool was used for each scenario (Supplementary Files 3A–C). Teams could achieve a maximum of 60 points with each scenario for various medical actions (e.g., adherence to treatment algorithms according to European Resuscitation Council (17) and team behavior, including crisis resource management principles (18), team reflections (19), STOP sequences, “10 s for 10 min” (18) and effective communication strategies such as “closed-loop” communication (20, 21). The tool was based on evaluation tools used for previous simulation competitions (personal communication RML) and adapted after current evaluation tools for the team behavior part (22). The team with the highest number of points achieved in the final scenario was named the winner. The jurors had prepared a pediatric resuscitation guideline quiz in case two teams had the same number of points to appoint the winning team, which was ultimately to be presented for educational purposes only. For all three simulated cases, the organizing committee (RML, one pediatric expert from Switzerland, Austria, and Germany) and jurors performed a short medical debriefing for all participating teams and the audience. Teamwork aspects were debriefed in a separate virtual debriefing session with each team and without an audience the day after the competition to maintain psychological safety. A complete description of the competition and the differences between the two simulation modalities can be found in Table 1.@@[23, 24]

**Table 1:** Description of the simulation competition.

	Avatar simulation	Distance simulation
Teams	One coach (pediatric specialist), one nurse, three pediatric specialists in training	One coach (pediatric specialist), one nurse, three pediatric specialists in training
Scenario	We ran the same scenario* (supraventricular tachycardia case) four times. Avatars acted solely to orders from the participating teams and were addressed by the colour of their scrubs (which acted as a name)	Two different scenarios* (drowning and status epilepticus) were run twice each for teams 1 + 2 and 3 + 4, respectively.
Technical details	Teams, jurors, and avatars joining a Zoom conference, avatar team and monitor pitched to Zoom screen using iSimulate software (23).	Teams, jurors, and audience joining a Zoom conference, participating team and monitor pitched to Zoom screen using SIMStation software (24) (own monitor, *n* = 2, operated by confederate) or iSimulate software (23) (*n* = 2, operated by organising team/juror).
Audience	None	Conference workshop participants (*n* = 12), observing the competition and participating in the feedback/medical debriefing session.
Organising committee	Jurors: RML (pediatric emergency medicine), located in Sweden, one juror located in Switzerland (pediatric emergency medicine) and 1 in Germany (paediatrics), respectively	Jurors: RML (pediatric emergency medicine), located in Sweden, one juror located in Switzerland (pediatric emergency medicine) and 1 in Germany (paediatrics), respectively
	Technical support: Pediatrics specialist, Austria, physically located in Vienna	Technical support: Pediatrics specialist, Austria, physically located in Vienna
Evaluation	60-point scale scoring sheet**, points and penalty points for various performed or omitted medical and non-medical tasks	60-point scale scoring sheet**, points and penalty points for various performed or omitted medical and non-medical tasks
Debriefing	15-minute presentation-based, interactive summary of medical aspects of each scenario for all participants and audience	15-minute presentation-based, interactive summary of medical aspects of each scenario for all participants and audience; separate teamwork-oriented virtual debriefing session with each team and without an audience the day after the competition

* The original scenarios are available on request by the authors.

** The original scoring sheets are provided as supplementary files 3a-c.

#### Questionnaire development

To develop a questionnaire to explore participants' opinions of avatar and distance simulation compared to conventional SBE, we used a three-round modified Delphi technique. The Delphi technique allows easy curriculum revision, as investigators can work at a distance with various target group representatives. It provides opinions from a broad range of experts to be consolidated into a manageable number of precise statements. This technique defines that “pooled intelligence” captures the collective view of stakeholders (25). The recourse was taken to five simulation education experts who were not part of the competition jury. RML drafted the first version of the questionnaire, which was composed of open and multiple-choice questions as well as questions using a Likert-like scale with 1 representing “Extremely little/low”, 2 “Very little/low”, 3 “Low/little”, 4 “Neutral/average”, 5 “Well/much”, and 6 “Very well/much”, and 7 “Extremely well/much”. All Delphi rounds were developed iteratively by consultation and feedback. In the first Delphi round, we used open-ended questions with the scope of prioritizing and putting across the most relevant survey questions and topics for avatar and distance simulation.

Participating stakeholders were asked to comment on the content, comprehensibility, grammar/spelling, completeness, and relevance of the survey items to suggest changes or decide whether the items should be included in the final competence list. All participating stakeholders were invited by e-mail to answer the questionnaire. An e-mail reminder was sent 14 days after the initial invitation. After completing the first round, the facilitator (RML) read all the answers to the open questions, edited, merged similar answers/suggestions, and grouped them into categories to compile the second-round questionnaire. All stakeholders participating in round 1 were invited by e-mail to the second round to rate each statement. The second round consisted of a repeat review of the edited survey items regarding content, comprehensibility, grammar/spelling, completeness, and relevance in this edited version. Items included in the questionnaire were again re-piloted, and final edits were made based on the feedback. In the third round, the penultimate list was again sent to all the stakeholders to comment on and sign. At the end of Delphi round 3 a consensus was reached, resulting in the final 32-item version of the survey (original questionnaire in German and English translation thereof, Supplementary Files 1, 2). The final online version was pilot tested for ease of completion and technical functioning by two German-speaking stakeholders to confirm its comprehensibility and the usefulness of the response options.

#### Simulation competition participant survey

After the workshop, all four participating teams were surveyed using the previously developed 32-item online questionnaire (Supplementary Files 1, 2). The survey link was sent to all potential study participants following the simulation competition, including a covering letter reiterating the study's goals. The questionnaire aimed to explore the participants' experience with avatar simulation and distance simulation. The survey was distributed electronically using the SurveyMonkey® tool (SurveyMonkey Inc, San Mateo, California, United States). As all participants who enrolled in the workshop were eligible for inclusion in the study, we used a non-probability convenience sample.

The advantages of avatar and distance simulation, based on the qualitative analysis of open-ended questions (see Data analysis), and limitations and barriers of both simulation modalities, were declared as our primary outcomes.

The secondary outcomes included satisfaction with avatar and distance simulation, ability to immerse into and psychological safety with the avatar and distance simulation modes, availability of non-verbal information, preference for distance vs. presential simulation modalities, and preference for virtual or hybrid vs. presential simulation in the future.

Participant demographics [age, gender, home country, simulation experience in years, previous virtual simulation experience (distance, avatar, telesimulation, or virtual reality simulation)] were recorded. We also collected the study participants' experiences regarding audiovisual broadcasting technology.

#### Data analysis

We used SPSS v26 (IBM, New York, United States) to analyze quantitative data. We performed a descriptive analysis of the survey data. Categorical variables were described as absolute (n) and relative frequencies (%). Missing answers are accounted for by listing the total absolute number for each item (different from 20) where not all 20 respondents answered a question. Continuous variables were described using median and 25th–75th quartiles. Individual items were assessed for normal distribution with the Shapiro–Wilks test and visual assessment of residuals and Q–Q Plots. Due to the nature of the data and the small sample, we used the Mann–Whitney *U*, Kruskal–Wallis or Wilcoxon signed-rank tests for continuous variables and the Chi-square test or Fisher's exact test for categorical variables. An *a priori* probability of less than 0.05 was considered statistically significant. For reliability testing of the survey, internal consistency was evaluated with Cronbach's alpha (Cronbach's alpha = 0.7) (JB-E). We considered a Cronbach's alpha of ≥0.7 as reliable (26).

The qualitative analysis of open-ended questions was performed by two authors (JB-E and RML) using an inductive approach and a thematic content analysis (27). JB-E performed answer reduction, and RML cross-checked answers. We used open coding to check for common themes and categories of open-ended questions. Open codes were re-analyzed for duplications and overlapping themes. Final code verification was attained through peer debrief.

## Results

Four teams (Germany *n* = 1, Austria *n* = 3, total participants *n* = 20) participated in the study. Participant characteristics are presented in Table 2.

**Table 2:** Participant demographics.

	Total (*n* = 20)
Age [years, Median (Q25–Q75)]	34.0 (29.0–41.0)
Female gender (*n*, %)	11 (55.0)
**Country of workplace**	
Austria	11 (55.0)
Germany	7 (35.0)
No answer	2 (10.0)
Simulation experience [years, Median (Q25–Q75)]	4.0 (2.5–7.5)
Previous experience in virtual/distance simulation	2 (10%)

### Survey results – technical aspects

Most participants attended the sessions using their computer or tablet with a camera and microphone. Only a small percentage (20%, *n* = 4/20) used the mobile phone. Regarding connection issues during the sessions, 40% (*n* = 8/20) of participants reported audio problems, and 25% (*n* = 5/20) reported video problems during the live broadcast. Most frequently reported problems included the inability to read the information on the screen (57.1%, *n* = 8/14), the commands to the avatars not being heard or misinterpreted (28.6%, *n* = 4/14) and frozen video transmission (14.2%, *n* = 2/14). These issues led to simulation delays on four occasions (20%). There were no differences between males and females in reporting audio (*p* = 0.619) or video (*p* = 0.371) issues. Participants managed to immerse themselves well in the avatar simulation [5.0 (3.0–5.0)] but received little non-verbal information from the avatars [5.0 (3.0–5.0)].

### Survey results – comparison of distance, avatar and face to face simulation

Participants’ satisfaction with distance simulation was higher than with avatar simulation, but this difference was not statistically significant (*z* = −1.00, *p* = 0.317). We found no difference in the generally high rating of psychological safety in both simulation types (Table 3). However, compared to presential simulation, only 20% of participants (*n* = 4/20) believed that avatar simulation offered more psychological safety, while distance simulation was considered psychologically safe in 30% (*n* = 6/20) of cases. There were no significant differences between men and women for satisfaction with the simulation (avatar: *p* = 0.388; distance: *p* = 0.313), for psychological safety (avatar: *p* = 0.755; distance: *p* = 0.0713) and for the ability for immersion (*p* = 0.662) and reception of non-verbal cues (*p* = 0.628) in the avatar simulation. Overall, the Likert-scale items in the survey showed an internal consistency of *α* = 0.763. Reported advantages and challenges for each type of simulation compared with traditional presential simulation are listed in Table 4. Regarding preferences for different future simulation types, responders did not show any interest in changing to avatar simulation only (*n* = 8/20, 40%). They also seemed to prefer presential simulation to distance simulation (*n* = 7/20, 35%) but showed interest in having presential and avatar/distance options or hybrid options available.

**Table 3:** Satisfaction and psychological safety with avatar simulation and distance simulation.

	Avatar Simulation	Distance Simulation	*p*-value*
Satisfaction [Median (Q25–Q75)]	5.0 (4.0–6.0)	6.0 (5.0–6.0)	0.317
Psychological Safety [Median (Q25–Q75)]	5.0 (4.0–6.0)	5.0 (5.0–6.0)	0.480

* Wilcoxon signed-rank test. Satisfaction and psychological safety were rated on a Likert-scale from 1 (Extremely little) to 7 (Extremely much) for satisfaction, and 1 (Extremely low) to 7 (Extremely high) for psychological safety, respectively.

**Table 4:** Reported advantages and challenges with avatar and distance simulation.

	Avatar simulation	Distance simulation
Advantages	• No travel distances (*n* = 10/30, 33.3%) • Lower costs (*n* = 9/30, 30.0%) • Less time required (*n* = 9/30, 30.0%) • Less exposure to other participants or educators (*n* = 1/30, 3.3%) • Other (*n* = 1/30, 3.3%)	• No travel distances (*n* = 14/44, 31.8%) • Lower costs (*n* = 10/44, 22.7%) • Less time required (*n* = 7/44, 15.9%) • Less exposure to other participants or educators (*n* = 2/44, 4.5%) • Simulation in real working environment (*n* = 10/44, 22.7%) • Other (*n* = 1/44, 2.2%)
Challenges	• Technical problems (*n* = 9/38, 23.7%) • Streaming delays (*n* = 5/38, 13.2%) • Fewer non-verbal cues (*n* = 9/38, 23.7%) • Spatial distance from team (*n* = 9/38, 23.7%) • Feeling “hands tied” (*n* = 4/38, 10.5%) • Other (*n* = 2/38, 5.2%)	• Technical problems (*n* = 16/37, 43.2%) • Technology time delays (*n* = 8/37, 21.6%) • Spatial distance from team (*n* = 12/37, 32.4%) • Other (*n* = 1/37, 2.7%)

When asked about psychological safety, respondents mentioned that during presential simulation there was easier communication and engagement in teamwork, more direct contact with other participants, less hesitancy to talk about emotions, easier understanding of non-verbal cues, and more authentic debriefing. As for avatar simulation, limited involvement of trainers, a reduced feeling of being examined, less shame when giving wrong answers, and the physical distance between trainers and participants were considered beneficial for psychological safety. Statements were similar for distance simulation, including being less on display, suffering less from exam stress, valuing the training in a familiar working environment and trainers feeling more like supervisors.

Responses related to general learning experiences with virtual simulation are summarized in Table 5.

**Table 5:** Reported reasons why different types of simulation offer psychological safety in different ways and reported learning experiences from distance simulation.

**Reported reasons why presential simulation enhances psychological safety:** Easier communication and engagement in teamwork Easier to receive direct and complete feedback More direct and immediate contact with the other participants Getting to know the other participants personally Less inhibition to talk about emotions Easier understanding of non-verbal cues More effective and authentic debriefing with participants and debriefer physically in the same room, (*full*) debriefing directly after the scenario Atmosphere is more authentic/ direct and less anonymous “No unknown number of (invisible) spectators” (n=2) (*This was the case in this simulation competition*) “This may only be a subjective psychological safety” **Reported reasons why avatar simulation enhances psychological safety:** Trainers may have a better overview from a distance, may be less involved in the simulation Less feeling of shame when giving wrong answers The physical distance between trainers and participants conveys a feeling of security Less exam feeling **Reported reasons why distance simulation enhances psychological safety:** Simulation in familiar working environment “Trainer feels more like a supervisor” Less feeling of being on display Less exam stress **Reported specific learning experiences with distance and avatar simulation:** Preparation and possibility of technical equipment/technology Possibility of interaction with different involved persons who do not need to be physically in the same room More positive experience than expected, unexpectedly effective team training More challenging assessment of non-medical aspects in distance, as compared to F2F simulation New (*virtual*) situation, flexibility Stresses the relevance of good communication, repeat assessments and keeping calm "First sim for me in a while so grateful for the experience" Good opportunity to train team-leadership (*with a focus on clear instructions and structured patient assessment*) Excellent opportunity for students (*in current and future contexts*) “Interesting experience, for me more like watching a video or personal algorithm, less like team training” It is difficult to lead the avatars step for step, but it is good for communication training

* In parentheses, explanatory notes by the authors.

## Discussion

Participants indicated a high overall satisfaction with avatar and an even higher, although statistically non-significant, appreciation of distance simulation in this Central European survey study. Our hypothesis that there would be distinct but specific advantages of avatar and distance simulation compared to standard SBE was corroborated.

### Strengths of avatar and distance simulation

The surveyed participants mentioned several decisive strengths of virtual simulation education in the inaugural virtual simulation competition facilitated by Netzwerk Kindersimulation, e.V. (14). The elimination of travel distances and associated lower costs and less time spent attending the education activity were the most frequently reported items. Although mentioned least commonly, reduced self-exposure to other participants or educators seemed advantageous. Previous participant satisfaction survey-based studies described telesimulation as a good substitute for presential SBE and the fact that they felt more engaged and encouraged to think critically (28). Virtual simulation offers further profits. For example, permitting remote specialists or colleagues to participate as educators or debriefers on a topic they are experts in and thereby offers widened expertise to the participants on the one hand, and peer coaching or debriefing of the debriefing among experts on the other hand (29). It has been recommended that simulation educators regularly get feedback on their debriefing performance, which could be facilitated more easily through virtually attending and more experienced debriefing colleagues (21). This concept, called telementoring, has resulted in high-quality debriefings involving remote and local instructors to facilitate effective debriefing during telesimulation (29).

It has been described that simulation integrated into the actual working environment (*in situ simulation*) entails the chance to improve reliability and safety in high-risk areas. In addition, it allows the identification of latent clinical and system threats to patient safety. It provides realism through deliberate practice and integration of interdisciplinary and interprofessional teamwork skills in the time-pressured clinical context. Ultimately, it leads to change in clinical care systems and improved clinical outcomes (30).

Another reported unique benefit of avatar simulation included a positive learning experience due to the more pronounced and newly learned relevance of direct, clear, and structured communication and team leadership when leading the avatars. To our knowledge, this effect has not been previously reported. Whilst the evidence base for this effect is still in its infancy, the same effect has been described with blindfold team training and closed-loop communication. Like leading a team of avatars at a distance, blindfold training is hands-off. It requires critical thinking skills, a conceptual framework, and highly specific and transparent communication within the team to prevent communication errors (31).

Regarding distance simulation in particular, attendance of the simulation event in the own familiar working environment was a commonly mentioned win. Interestingly, one participant stated that the passive attendance of a scenario managed by a competitor team felt like “watching a video or personal algorithm training, less like team training”. While this (individual) impression could be seen as a potential limitation of avatar simulation for team training (due to limited immersion into the avatar environment and suspension of disbelief), it may simply reflect the need to develop this simulation modality further and implement it purposefully.

### Avatar simulation

An avatar is a concept that originated within Hinduism that signifies a deity's material appearance or incarnation. In computing, an avatar is a graphical representation of a user in a virtual environment (32). It may take either a two-dimensional form as an icon (also known as a profile picture) or a three-dimensional form, e.g., in games or virtual worlds (33). Avatars originated in the world of gaming as popular components of virtual reality (VR). VR formats are increasingly used in healthcare for education and patient distraction preoperatively or during painful procedures (34).

While avatar research is still in its early stages, evidence suggests that those who receive tailored guidance and advice from these virtual agents appear to have better physical and psychosocial outcomes. One explanation is that the digital characters can be customized for cultural, social, and other user preferences (35). Avatars are also being progressively used in medical simulations (36). Immersive, three-dimensional worlds have been created in VR, which may even incorporate multisensory feedback to ensure students pick up essential skills and applications in different healthcare contexts (37, 38). Simulation participants in our study mainly were “novices” regarding the distance simulation construct, whilst it is assumed that they are competent in managing pediatric emergencies. Thus, a lack of concept familiarity may partly explain that most participants would have preferred presential simulation. Motivation to engage in virtual simulation also likely varies from actual patient care. Learners may choose a faster path to the perceived correct answer or be willing to perform educated guessing with less information in a low-stakes, no-patient-harmed environment (39), e.g., telesimulation. Previously reported profits of telesimulation are improved interinstitutional networking and collaboration and rapid dissemination of new medical contents (5).

### Face to face simulation

Presential simulation was the gold standard before the COVID-19 pandemic, and we expect this simulation mode to remain a mainstay of modern healthcare education and training. The apparent advantages with presential simulation indicated by the participants included more accessible communication and engagement in teamwork, mainly through non-verbal cues and capture of emotions, more direct contact with other participants and educators, and more authentic debriefing. Moreover, presential simulation requires less costly and time-consuming technical preparation efforts by the facilitators. Some avenues worth exploring in the future comprise the extent to which virtual simulation is comparable to traditional presential simulation, more targeted exploration of potential benefits, e.g., economic savings, time conservation and standardization of scenarios. Although our study did not evaluate whether distance simulation is equivalent to traditional presential simulation in assessing candidates' practical/learning skills, one can envision applying advanced virtual simulation technology to alleviate some of the barriers encountered in the current process. Accordingly, Abulfaraj et al. found no difference in learning outcomes after VR and high-fidelity manikin-based simulation training (40).

In addition to reducing travel distances and costs, virtual simulations can be administered from any remote location with computer access and at any time of day. Thus, simulations could be completed while on remote rotations or at a remote testing site, travelling or at home, across different time zones and countries. Many aspects of virtual simulation require exploration before such technology can be appropriately implemented for general use. Faculty perceptions and experiences need to be evaluated. Additionally, this format must be assessed for limitations regarding reliability, interobserver agreement, and available outcomes in formative and evaluative settings. In our study, participant feedback regarding further willingness to participate in distance simulation was overwhelmingly positive. In this study, many participants would have desired more interaction with the avatar, especially non-verbally and within the scenario. With currently available animation and programming capabilities, we can improve future simulations. Transition to automated scenarios without a real-life proctor could be achieved by applying artificial intelligence (41).

### Psychological safety - benefits

Likewise, participants perceived psychological safety as equally high with both virtual simulation types. Psychological safety is crucial for the successful use of SBE. It can be described as people's perception of the consequences of taking interpersonal risks (e.g., speaking up, asking questions, disclosing thoughts and mental frames) in contexts such as a workplace or an educational setting (42, 43).

Interestingly, participants felt that simulation at a distance from the trainers added a feeling of security and less exam stress, feeling of being on display or shame than presential simulation. These comments certainly underline the paramount importance of carefully setting the ground for psychological safety before each training, whether presential or virtual (43).

### Challenges with avatar and distance simulation

In contrast, propounded challenges by the participants included technical problems (23.7% with avatar and 43.2% with distance simulation) and the fact that the nature of virtual simulation incurred fewer non-verbal cues (23.7%) for avatar simulation and a spatial distance from the team (23.7% for avatar and 32.4% for distance simulation). Likewise, a previous learner satisfaction survey based study found poor ratings for audio quality (5.22, 6.63 and 5.8 on a 10-point Likert scale for the statement “I could hear the facilitator and other participants clearly”) during telemedical resident education (28), and technical issues related to network connectivity or sound quality during telesimulation for medical students neonatal resuscitation training in 75% (8). In our setting, facilitation of the virtual simulation competition relied entirely on the technical support of one institution without a backup institution. The encountered technical impediments can likely be eliminated with upgraded hardware and system capabilities. Furthermore, avatar simulation participants felt their “hands were tied” (10.5%). Contrarily, the occurrence of connection issues was relatively low, but some participants reported streaming and communication problems.

### Psychological safety – obstacles

The presence of spectators (*n* = 12), a delayed full debriefing, lack of direct contact with team members (avatar simulation) and a specific inhibition to discuss emotions during virtual simulation were reported as disadvantageous. Again, these comments highlight the importance of ensuring a high degree of psychological safety before the education activity by, e.g., underlining confidentiality and attributing enough time for debriefing (43).

Only 10% of the competition participants had previously participated in a virtual simulation. Nevertheless, participants managed to immerse themselves well in the avatar simulation, a new simulation environment for most participants, even though they reported restricted reception of non-verbal information from the avatars.

Despite the discussed profits and satisfaction with virtual simulation, none of the respondents wanted to convert to avatar simulation only (*n* = 8/20, 40%) or seemed to prefer presential simulation to distance simulation (*n* = 7/20, 35%). However, they showed an interest in having future hybrid presential and avatar/distance options available.

### General learning experience

Interestingly, most participants stated that especially avatar simulation offered a new and unexpectedly effective opportunity to specifically train effective communication, structured patient assessment and leadership skills. Both avatar and distance simulation formats generally provided the opportunity to participate in any simulation training at all, certainly underlining the beneficial win of equity for both remotely located medical and nursing students and health care providers and trainers (29).

### Limitations of the study

We acknowledge a small sample size in our study, where we investigated the preferences and opinions of a relatively small group of selected subjects in a very particular environment. Additionally, a subset of answers was missing due to a few incompletely answered questionnaires. Also, the avatar simulation was facilitated at a single academic training site. This research is based on participants' opinions, judgements, statements, and viewpoints that are not conclusive or scientific evidence compared to research data. All these factors may limit the generalizability of our results.

Furthermore, we investigated the feasibility of administering a virtual simulation in a competition setting; however, we did not evaluate the effectiveness of virtual simulation in assessing participants relative to a standard presential simulation. We did not specifically investigate the ability to immerse into distance simulation as we considered the *in situ* setting familiar to the participants but with the assessor/trainer at a distance. The simulation scoring tool used in this study was modified from the evaluation tools used in conventional simulation. From an educator's point of view, current evaluation tools might not be relevant to the new virtual environment. Thus, evaluation tools for virtual might need to be updated, or faculty may need to develop new tools specifically designed for the characteristics of distance simulation. We collected previous simulation experience but not experience in years in the clinical setting. We can, therefore, only assume that the trainees (4 × 3 junior trainees) had less experience than the specialists (4 × 1 senior physician), possibly causing cognitive bias.

## Conclusion

The results from this small but innovative pilot study will inform simulation educators about target group reported advantages and challenges of avatar and distance simulation modalities both in competition and training settings. While we fully acknowledge and emphasize the value of presential SBE, our findings suggest avatar-based simulation formats as a promising learning tool for targeted communication and leadership skills training for medical students and interdisciplinary and interprofessional teams in current and future education beyond the pandemic.

Although these findings may not be conclusive, they may undoubtedly inform future studies exploring the challenges and opportunities of different virtual simulation modalities and studies examining the experience and the degree of psychological safety in the virtual simulation context more extensively.

To date, presential training remains the gold standard of simulation-based education. However, virtual simulation training modalities will remain relevant for maintaining SBE during COVID-19 and other pandemics, forcing educators and learners to adhere to social distancing requirements while aiming to continue essential training activities.

## Data Availability

The raw data supporting the conclusions of this article will be made available by the authors, without undue reservation.

## References

[1] Zhu N Zhang D Wang W Li X Yang B Song J A novel coronavirus from patients with pneumonia in China, 2019. N Engl J Med. (2020) 382(8):727–33. 10.1056/NEJMoa2001017 31978945 PMC7092803

[2] Dedeilia A Sotiropoulos MG Hanrahan JG Janga D Dedeilias P Sideris M. Medical and surgical education challenges and innovations in the COVID-19 era: a systematic review. In Vivo. (2020) 34(3 suppl):1603–11. 10.21873/invivo.11950 32503818 PMC8378024

[3] Cook DA Hatala R Brydges R Zendejas B Szostek JH Wang AT Technology-enhanced simulation for health professions education: a systematic review and meta-analysis. JAMA [Internet]. (2011) 306(9)978–88. 10.1001/jama.2011.1234 21900138

[4] Mundell WC Kennedy CC Szostek JH Cook DA. Simulation technology for resuscitation training: a systematic review and meta-analysis. Resuscitation. (2013) 84(9):1174–83. 10.1016/j.resuscitation.2013.04.016. Available at: https://linkinghub.elsevier.com/retrieve/pii/S0300957213002463 (cited Jan 6, 2022). 23624247

[5] McCoy CE Sayegh J Alrabah R Yarris LM. Telesimulation: an innovative tool for health professions education. Yarris LM, editor. AEM Educ Train. (2017) 1(2):132–6. 10.1002/aet2.10015 30051023 PMC6001828

[6] von Lubitz DKJE Carrasco B Gabbrielli F Ludwig T Levine H Patricelli F Transatlantic medical education: preliminary data on distance-based high-fidelity human patient simulation training. Stud Health Technol Inform. (2003) 94:379–85. PMID: 15455929

[7] Jain A Agarwal R Chawla D Paul V Deorari A. Tele-education vs classroom training of neonatal resuscitation: a randomized trial. J Perinatol. (2010) 30(12):773–9. 10.1038/jp.2010.42 20357810

[8] Mileder LP Bereiter M Wegscheider T. Telesimulation as a modality for neonatal resuscitation training. Med Educ Online. (2021) 26(1):1892017. 10.1080/10872981.2021.1892017 33602053 PMC7899687

[9] Pennington KM Dong Y Coville HH Wang B Gajic O Kelm DJ. Evaluation of TEAM dynamics before and after remote simulation training utilizing CERTAIN platform. Med Educ Online. (2018) 23(1):1485431. 10.1080/10872981.2018.1485431 29912676 PMC6008595

[10] Lioce L, editor. Healthcare simulation dictionary [Internet]. Second. Agency for Healthcare Research and Quality (2020). Available at: https://www.ahrq.gov/patient-safety/resources/simulation/terms.html (cited Jan 6, 2022)

[11] Cobb SVG Nichols S Ramsey A Wilson JR. Virtual reality-induced symptoms and effects (VRISE). Presence Teleoperators Virtual Environ. (1999) 8(2):169–86. 10.1162/105474699566152. Available at: https://direct.mit.edu/pvar/article/8/2/169-186/18225 (cited Jan 6, 2022).

[12] Nichols S Patel H. Health and safety implications of virtual reality: a review of empirical evidence. Appl Ergon. (2002) 33(3):251–71. 10.1016/S0003-6870(02)00020-0 12164509

[13] Wagner M Jaki C Löllgen RM Mileder L Eibensteiner F Ritschl V Readiness for and response to coronavirus disease 2019 among pediatric healthcare providers: the role of simulation for pandemics and other disasters*. Pediatr Crit Care Med. (2021) 22(6):e333–8. 10.1097/PCC.0000000000002649 33350800 PMC8162220

[14] Netzwerk Kindersimulation e.V. https://www.netzwerk-kindersimulation.org.

[15] World Medical Association. World medical association declaration of Helsinki: ethical principles for medical research involving human subjects. JAMA. (2013) 310(20):2191–4. 10.1001/jama.2013.281053 24141714

[16] https://www.gnpi2021.de.

[17] Van de Voorde P Turner NM Djakow J de Lucas N Martinez-Mejias A Biarent D European Resuscitation council guidelines 2021: paediatric life support. Resuscitation. (2021) 161:327–87. 10.1016/j.resuscitation.2021.02.015 33773830

[18] Rall M Gaba D. Human performance and patient safety. In: Millar R, editors. Miller's anaesthesia. Philadelphia: Elsevier (2005). p. 3021–72.

[19] Schmutz JB Kolbe M Eppich WJ. Twelve tips for integrating team reflexivity into your simulation-based team training. Med Teach. (2018) 40(7):721–7. 10.1080/0142159X.2018.1464135 29703126

[20] El-Shafy IA Delgado J Akerman M Bullaro F Christopherson NAM Prince JM. Closed-Loop communication improves task completion in pediatric trauma resuscitation. J Surg Educ. (2018) 75(1):58–64. 10.1016/j.jsurg.2017.06.025. Available at: https://linkinghub.elsevier.com/retrieve/pii/S1931720417300387 (cited Jan 6, 2022). 28780315

[21] Löllgen R Mileder L Wagner M Bibl K Paulun A Rupp J Recommendations of the Netzwerk Kindersimulation e.V. for the implementation of paediatric simulation-based team trainings (2020). Available at: https://www.netzwerk-kindersimulation.org/qualitaetskriterien/. 10.3390/children10061068 PMC10297481 37371299

[22] Thomas EJ Sexton JB Lasky RE Helmreich RL Crandell DS Tyson J. Teamwork and quality during neonatal care in the delivery room. J Perinatol Off J Calif Perinat Assoc. (2006) 26(3):163–9. 10.1038/sj.jp.7211451 16493432

[23] https://www.3bscientific.com/isimulate,isim.html.

[24] https://www.simstation.com/de.

[25] de Villiers MR de Villiers PJT Kent AP. The Delphi technique in health sciences education research. Med Teach. (2005) 27(7):639–43. 10.1080/13611260500069947 16332558

[26] Bland JM Altman DG. Statistics notes: Cronbach’s alpha. BMJ. (1997) 314(7080):572–572. 10.1136/bmj.314.7080.572 9055718 PMC2126061

[27] Chapman AL Hadfield M Chapman CJ. Qualitative research in healthcare: an introduction to grounded theory using thematic analysis. J R Coll Physicians Edinb. (2015) 45(3):201–5. 10.4997/jrcpe.2015.305 26517098

[28] Patel SM Miller CR Schiavi A Toy S Schwengel DA. The sim must go on: adapting resident education to the COVID-19 pandemic using telesimulation. Adv Simul. (2020) 5(1):26. 10.1186/s41077-020-00146-w PMC7522907 32999738

[29] Gross IT Whitfill T Auzina L Auerbach M Balmaks R. Telementoring for remote simulation instructor training and faculty development using telesimulation. BMJ Simul Technol Enhanc Learn. (2021) 7(2):61–5. 10.1136/bmjstel-2019-000512 35520375 PMC8936763

[30] Patterson M Blike G Nadkarni V. In situ simulation: Challenges and results. In: Henriksen K Battles J Keyes M, editors. Advances in patient safety: New directions and alternative approaches. Rockville, MD: Agency for Healthcare Research and Quality (United States) (2008). (Vol. 3: Performance and Tools). Available at: https://www.ncbi.nlm.nih.gov/books/NBK43682/21249938

[31] Ahmed R Hughes K Hughes P. The blindfolded code training exercise. Clin Teach. (2018) 15(2):120–5. 10.1111/tct.12639 28382769

[32] O’Connor S. Virtual reality and avatars in health care. Clin Nurs Res. (2019) 28(5):523–8. 10.1177/1054773819845824 31064283

[33] Lessig L. Code: And other laws of cyberspace. Nachdr. New York: The Perseus Books Group (2002). 297.

[34] Esposito C Autorino G Iervolino A Vozzella EA Cerulo M Esposito G Efficacy of a virtual reality program in pediatric surgery to reduce anxiety and distress symptoms in the preoperative phase: a prospective randomized clinical trial. J Laparoendosc Adv Surg Tech. (2022) 32(2):197–203. 10.1089/lap.2021.0566 34962159

[35] Shafii T Benson SK Morrison DM Hughes JP Golden MR Holmes KK. Results from e-KISS: electronic-KIOSK intervention for safer sex: a pilot randomized controlled trial of an interactive computer-based intervention for sexual health in adolescents and young adults. Bellamy SL, editor. PLoS One. (2019) 14(1):e0209064. 10.1371/journal.pone.0209064 30673710 PMC6343886

[36] Umoren R Bucher S Hippe DS Ezenwa BN Fajolu IB Okwako FM eHBB: a randomised controlled trial of virtual reality or video for neonatal resuscitation refresher training in healthcare workers in resource-scarce settings. BMJ Open. (2021) 11(8):e048506. 10.1136/bmjopen-2020-048506 34433598 PMC8390148

[37] Skiba DJ. Nursing education 2.0: a second look at second life. Nurs Educ Perspect. (2009) 30(2):129–31. PMID: 19476080

[38] McCallum J Ness V Price T. Exploring nursing students’ decision-making skills whilst in a second life clinical simulation laboratory. Nurse Educ Today. (2011) 31(7):699–704. 10.1016/j.nedt.2010.03.010. Available at: https://linkinghub.elsevier.com/retrieve/pii/S0260691710000663 (cited Jan 6, 2022 ). 20807671

[39] Bond WF Lynch TJ Mischler MJ Fish JL McGarvey JS Taylor JT Virtual standardized patient simulation: case development and pilot application to high-value care. Simul Healthc J Soc Simul Healthc. (2019) 14(4):241–50. 10.1097/SIH.0000000000000373. Available at: https://journals.lww.com/01266021-201908000-00006 (cited Jan 6, 2022). 31116172

[40] Abulfaraj MM Jeffers JM Tackett S Chang T. Virtual reality vs. high-fidelity mannequin-based simulation: A Pilot randomized trial evaluating learner performance. *Cureus* (2021). Available at: https://www.cureus.com/articles/64747-virtual-reality-vs-high-fidelity-mannequin-based-simulation-a-pilot-randomized-trial-evaluating-learner-performance (cited Jan 9, 2022). 10.7759/cureus.17091 PMC8432415 34527478

[41] Danforth DR Procter M Chen R Johnson M Heller R. Development of virtual patient simulations for medical education. J Virtual Worlds Res. (2009) 2(2):4–11. 10.4101/jvwr.v2i2.707

[42] Edmondson A Lei Z. Psychological safety: the history, renaissance, and future of an interpersonal construct. Annual Rev Org Psyc Organ Behav. (2014) 1:23–43. 10.1146/annurev-orgpsych-031413-091305

[43] Kolbe M Eppich W Rudolph J Meguerdichian M Catena H Cripps A Managing psychological safety in debriefings: a dynamic balancing act. BMJ Simul Technol Enhanc Learn. (2020) 6(3):164–71. 10.1136/bmjstel-2019-000470 35518370 PMC8936758

