# Supplementary material for: Avatar and distance simulation as a learning tool – virtual simulation technology as a facilitator or barrier? A questionnaire-based study on behalf of Netzwerk Kindersimulation e.V
Source: Front Pediatr. 2022 Oct 26;10:853243. doi: 10.3389/fped.2022.853243 (PMC9644191; doi:10.3389/fped.2022.853243)
Supplement: Supplementary file 1 [file Datasheet1.pdf]

Virtuelle Simulation als Lernwerkzeug - Technologie als Erleichterung oder Barriere?

Teilnehmerbefragung nach Avatar-und Fernsimulationsmeisterschaften organisiert durch das Netzwerk Kindersimulation e.V. während der GNPI Jahrestagung 2021

Demographics

1. Dein Alter
2. Dein Geschlecht
3. In welchem Land arbeitest Du?
4. Deine Simulationserfahrung in Jahren
5. Vorherige Erfahrung mit virtueller Simulation
    a. Ja
    b. Nein

Teil 1. Avatar-Simulation (=Avatar Team-Mitglieder und Teamleader räumlich distanziert)

6. Wie hat Dir die Avatar-Simulation insgesamt gefallen? (bitte die zutreffendste Antwort ankreuzen)

Extrem wenig	Sehr wenig	Wenig	Durchschnittlich	Gut	Sehr gut	Extrem gut
1	2	3	4	5	6	7

7. Gab es **Audio-Probleme** bei der Live-Übertragung durch die Verbindungsqualität des Internets?
    a. Ja
    b. Nein

8. Gab es **Video-Probleme** bei der Live-Übertragung durch die Verbindungsqualität des Internets?
    a. Ja
    b. Nein

9. Falls es Audio-Video Probleme gab, hat die Verzögerung die Simulation beeinträchtigt?
    a. Ja
    b. Nein
    c. Es gab keine Verzögerung

10. Falls die Simulation beeinträchtigt war, warum? (Mehrfachantwort möglich)
    a. Die Befehle an die Avatare wurden von den Avataren nicht gehört
    b. Die Antworten der Avatare wurden von den Teilnehmern nicht gehört
    c. Die Videoübertragung war eingefroren
    d. Man konnte nicht sehen, was auf dem Monitor zu lesen war
    e. Anderes (bitte beschreiben):

11. Wie gut gelang es Dir, in die Avatar-Simulation einzutauchen? "*suspension of disbelief*" and *«buy-in»?* (Bitte die zutreffendste Antwort ankreuzen)

Extrem wenig	Sehr wenig	Wenig	Durchschnittlich	Gut	Sehr gut	Extrem gut
1	2	3	4	5	6	7

(z.B. Extrem gut =7 = fühlte sich an, als wäre man im selben Raum gewesen, versus Extrem wenig = 1 = fühlte sich an, als hätte man ein Video gesehen)

12. Wie viel **nonverbale Information** *(= Information, die sich nicht auf eine sprachliche Informationsvermittlung stützt, sondern über alle Sinne kommuniziert wird z.B. durch* ***Gesten und Körperhaltung,*** *Geruch, Geschmack)* hast Du von den Avataren erhalten? (Bitte die zutreffendste Antwort ankreuzen)

Extrem wenig	Sehr wenig	Wenig	Durchschnittlich	Viel	Sehr viel	Extrem viel
1	2	3	4	5	6	7

13. Bitte nenne **Vorteile der Avatar-Simulation** im Vergleich zu Präsenz-Simulation für diesen Wettbewerb. (Mehrfachantwort möglich)
    a. Kein Anreiseweg
    b. Geringere Kosten für mich
    c. Weniger Zeitaufwand
    d. Ich kann im Pyjama teilnehmen
    e. Ich bin weniger sichtbar exponiert
    f. Andere (freie Kommentare):
    g. Keine

14. Bitte nenne **Herausforderungen bei der Avatar-Simulation** im Vergleich zu Präsenz-Simulation für diesen Wettbewerb: (Mehrfachantwort möglich)
    a. Technikprobleme (Audio-Video)
    b. Technik-bedingte zeitliche Verzögerung
    c. Weniger nonverbale Information
    d. Räumliche Distanz zum Team/zum Patienten
    e. «Hände gebunden»
    f. Andere (freie Kommentare):
    g. Keine

15. Wie bewertest du die **psychologische Sicherheit** *(= Vertrauensvolle Atmosphäre, in der alle Teammitglieder sich offen äußern können, ohne beschämt zu werden, abgewiesen zu werden, oder sonst wie negativ sanktioniert zu werden)* während der Avatar-Simulation? (bitte die zutreffendste Antwort ankreuzen)

Extrem niedrig	Sehr niedrig	niedrig	durchschnittlich	Hoch	Sehr hoch	Extrem hoch
1	2	3	4	5	6	7

16. Welche Modalität bietet Deiner Meinung nach eine höhere **psychologische Sicherheit**, Präsenz- oder Avatar-Simulation?
    a. Präsenz-Simulation
    b. Avatar-Simulation

17. Warum bietet Deiner Meinung nach Präsenz-Simulation höhere psychologische Sicherheit?
    a. (freie Kommentare)
    b. Nichtzutreffend

18. Warum bietet Deiner Meinung nach Avatar-Simulation höhere psychologische Sicherheit?
    a. (freie Kommentare)
    b. Nichtzutreffend

19. Würdest Du in Zukunft Avatar-Simulation gegenüber Präsenz-Simulation **im Wettkampfsetting** bevorzugen?
    a. Ja
    b. Nein
    c. Ich hätte gerne beides
    d. Ich hätte gerne eine Hybridlösung

20. Würdest Du in Zukunft Avatar-Simulations**training** gegenüber Präsenz-Simulations**training** bevorzugen?
    a. Ja
    b. Nein
    c. Ich hätte gerne beides

   d. Ich hätte gerne eine Hybridlösung (ein Teil der Teilnehmer, Trainer, Techniker oder Geräte sind "vor Ort" während andere sich an einem anderen, entfernten Standort befinden aber synchron in Simulationen interagieren)

21. Was nimmst du als grösste Lernerfahrung aus der Avatarsimulation mit?

Teil 2. Telesimulation (komplettes Team vor Ort, aber räumlich getrennt vom Debriefer)

22. Wie hat dir die Erfahrung mit der **Fern-Simulation über Zoom insgesamt gefallen**? (bitte die zutreffendste Antwort ankreuzen)

Extrem wenig	Sehr wenig	Wenig	durchschnittlich	gut	Sehr gut	Extrem gut
1	2	3	4	5	6	7

23. Welche Technologie hattest Du/hattet Ihr neben einer Internetverbindung vor Ort? (zutreffendes bitte ankreuzen, Mehrfachantwort möglich)
   a. Personal Computer/Laptop
   b. Kamera
   c. Tablet
   d. Mikrofon
   e. Mobiltelefon
   f. Andere (bitte nennen):
   g. Keine

24. Bitte nenne **Vorteile der Fern-Simulation** im Vergleich zu Präsenz-Simulation für diesen Wettbewerb: (Mehrfachantwort möglich)
   a. Kein Anreiseweg
   b. Geringere Kosten für mich
   c. Weniger Zeitaufwand
   d. Ich kann in meiner vertrauten Arbeitsumgebung simulieren
   e. Weniger Exposition
   f. Andere (freie Kommentare):
   g. Keine

25. Bitte nenne **Herausforderungen bei der Fern-Simulation** im Vergleich zu Präsenz-Simulation für diesen Wettbewerb: (Mehrfachantwort möglich)
   a. Mögliche Technikprobleme (Audio-Video)
   b. Technik-bedingte zeitliche Verzögerung
   c. Räumliche Distanz zum Debriefer
   d. Andere (freie Kommentare):
   e. Keine

26. Wie bewertest du die **psychologische Sicherheit** *(= vertrauensvolle Atmosphäre, in der alle Teammitglieder sich offen äußern können, ohne beschämt zu werden, abgewiesen zu werden, oder sonst wie negativ sanktioniert zu werden)* während der Fern-Simulation?
(bitte die zutreffendste Antwort ankreuzen)

Extrem niedrig	Sehr niedrig	niedrig	durchschnittlich	Hoch	Sehr hoch	Extrem hoch
1	2	3	4	5	6	7

27. Welche Modalität bietet Deiner Meinung nach höhere **psychologische Sicherheit**, Präsenz- oder Fern-Simulation?
   a. Präsenz-Simulation
   b. Fern-Simulation

28. Warum bietet Deiner Meinung nach Präsenz-Simulation höhere psychologische Sicherheit?
   a. (Freie Kommentare):
   b. Nichtzutreffend

29. Warum bietet Deiner Meinung nach Fern-Simulation höhere psychologische Sicherheit?

   a. (Freie Kommentare):
   b. Nichtzutreffend

30. Würdest Du in Zukunft Fern-Simulation gegenüber Präsenz-Simulation **im Wettkampfsetting** bevorzugen?
    a. Ja
    b. Nein
    c. Ich hätte gerne beides
    d. Ich hätte gerne eine Hybridlösung (ein Teil der Teilnehmer, Trainer, Techniker oder Geräte sind "vor Ort" während andere sich an einem anderen, entfernten Standort befinden aber synchron in Simulationen interagieren)

31. Würdest Du in Zukunft Fern-Simulations**training** gegenüber Präsenz-Simulations**training** bevorzugen?
    a. Ja
    b. Nein
    c. Ich hätte gerne beides
    d. Ich hätte gerne eine Hybridlösung

32. Was nimmst du als grösste Lernerfahrung aus der Fern-Simulation mit?

HERZLICHEN DANK!

NETZWERK
KINDERSIMULATION
