# Supplementary material for: Avatar and distance simulation as a learning tool – virtual simulation technology as a facilitator or barrier? A questionnaire-based study on behalf of Netzwerk Kindersimulation e.V
Source: Front Pediatr. 2022 Oct 26;10:853243. doi: 10.3389/fped.2022.853243 (PMC9644191; doi:10.3389/fped.2022.853243)
Supplement: Supplementary file 2 [file Datasheet2.pdf]

NETZWERK KINDERSIMULATION

Virtual simulation as a learning tool - technology facilitator or barrier?

Participant survey post avatar and distance simulation competition facilitated by Netzwerk Kindersimulation e.V. at the annual GNPI 2021 meeting

Demographics
1) your age
2) your gender
3) in which country do you work?
4) your simulation experience in years
5) previous experience with virtual simulation
   a. Yes
   b. No

PART 1. AVATAR SIMULATION (=Avatar team members and team leader spatially distanced)
6) How did you like the avatar simulation overall? (Please tick the most appropriate answer)

Extremely little	Very little	Little	Average	Much	Very much	Extremely much
1	2	3	4	5	6	7

7) Were there audio problems during the live broadcast due to the connection quality of the internet?
   a. Yes
   b. No

8) Were there video problems during the live broadcast due to the connection quality of the internet?
   a. Yes
   b. No

9) If there were audio-video problems, did the delay affect the simulation?
   a. Yes
   b. No
   c. There was no delay

10) If the simulation was affected, why? (Multiple answers possible)
   a. The commands to the avatars were not heard by the avatars
   b. The responses of the avatars were not heard by the participants
   c. The video transmission was frozen
   d. One could not see what was being read on the monitor
   e. Other (please describe):

11) How well did you manage to immerse yourself in the avatar simulation? "Suspension of disbelief" and "buy-in"? (Please tick the most appropriate answer)

Extremely little	Very little	Little	Not well, not little	well	Very well	Extremely well
1	2	3	4	5	6	7

(Extremely well = acted as if was in the same room =7 versus extremely little = acted as if watched a video=1)

12) How much non-verbal information (= Information that is not based on language but communicated through all senses, e.g., through gestures and posture, smell, taste) did you receive from the avatars? (Please tick the most appropriate answer)

Extremely little	Very little	Little	Not much not little	Much	Very much	Extremely much
1	2	3	4	5	6	7

13) Please list the advantages of avatar simulation compared to presential simulation for this competition. (Multiple answers possible)
a. No travel distances
b. Lower costs for me
c. Less time required
d. I can participate in my pajamas
e. I am less visibly exposed
f. Others (free comments):
g. None

14) Please list challenges in avatar simulation compared to presential simulation for this competition (Multiple answers possible)
a. Technical problems (audio-video)
b. Time delay due to technology
c. Less non-verbal information
d. Spatial distance from the team/patient
e. “Hands tied”
f. Others (free comments):
g. None

15) How would you rate the **psychological safety** (= trusting atmosphere in which all team members can openly express themselves without being shamed, rejected or otherwise negatively sanctioned) during the Avatar simulation? (Please tick the most appropriate answer)

Extremely low	Very low	Low	average	high	Very high	Extremely high
1	2	3	4	5	6	7

16) Which modality do you think offers higher psychological safety, presential or avatar simulation?
a. Presential simulation
b. Avatar simulation

17) Why do you think presential simulation offers higher psychological safety?
a. (Free comments)
b. Not applicable

18) Why do you think avatar simulation offers higher psychological safety?
a. (Free comments)
b. Not applicable

19) In the future, would you prefer avatar simulation to presential simulation in a competition setting?
a. Yes
b. No
c. I would like to have both
d. I would like a hybrid solution

20) Would you prefer avatar simulation training to face-to-face simulation training in the future?
a. Yes
b. No
c. I would like to have both
d. I would like to have a hybrid solution (some of the participants, trainers, technicians, or equipment are "on site" while others are at another, remote location but interacting synchronously in simulations).

21) What do you take away as the biggest learning experience from the avatar simulation?

PART 2. DISTANCE SIMULATION (Telesimulation = complete team on site, but spatially separated from the debriefer)

22) Overall, how did you like the experience of remote simulation via Zoom? (Please tick the most appropriate answer)

Extremely little	Very little	Little	Average	Much	Very much	Extremely much
1	2	3	4	5	6	7

23) What technology did you have besides an internet connection on site? (Please tick as appropriate, multiple answers possible)
a. Personal computer/laptop
b. Camera
c. Tablet
d. Microphone
e. Mobile phone
f. Other (please name):
g. None

24) Please list advantages of remote simulation compared to face-to-face simulation for this competition (Multiple answers possible)
a. No travel distances
b. Lower costs for me
c. Less time required
d. I can simulate in my familiar working environment
e. Less exposure
f. Other (free comments):
g. None

25) Please list challenges of remote simulation compared to face-to-face simulation for this competition (Multiple answers possible)
a. Possible technical problems (audio-video)
b. Time delay due to technology
c. Spatial distance to the debriefer
d. Other (free comments):
e. None

26) How do you rate the **psychological safety** (= Trusting atmosphere in which all team members can openly express themselves without being shamed, rejected or otherwise negatively sanctioned) during the remote simulation?
(Please tick the most appropriate answer)

Extremely low	Very low	Low	average	high	Very high	Extremely high
1	2	3	4	5	6	7

27) Which modality do you think offers higher psychological safety: face-to-face or remote simulation?
a. Presential simulation
b. Remote simulation

28) Why do you think presential simulation offers higher psychological safety?
a. (Free comments):
b. Not applicable

29) Why do you think remote simulation offers higher psychological safety?
a. (Free comments):
b. Not applicable

30) In the future, would you prefer remote simulation to presential simulation in the competition setting?
a. Yes
b. No
c. I would like to have both
d. I would like to have a hybrid solution (some of the participants, trainers, technicians, or equipment are "on site" while others are at another, remote location but interacting synchronously in simulations).

31) Would you prefer distance simulation training to face-to-face simulation training in the future?
a. Yes
b. No
c. I would like to have both
d. I would like a hybrid solution

32) Was nimmst du als grösste Lernerfahrung aus der Fern-Simulation mit?

THANK YOU VERY MUCH!

NETZWERK KINDERSIMULATION
