# Supplementary material for: Avatar and distance simulation as a learning tool – virtual simulation technology as a facilitator or barrier? A questionnaire-based study on behalf of Netzwerk Kindersimulation e.V
Source: Front Pediatr. 2022 Oct 26;10:853243. doi: 10.3389/fped.2022.853243 (PMC9644191; doi:10.3389/fped.2022.853243)
Supplement: Supplementary file 3 [file Datasheet3.pdf]

NETZWERK KINDERSIMULATION

Team: **Szenario: SVT**

ATEMWEGE		
Atemwege Check	☐	
Atemwege frei legen	☐	

Punkte: /2

ATMUNG		
Atemfrequenz checken	☐	
$Sa0_2$% messen/Monitoring	☐	
Auskultation	☐	
Atemarbeit/respiratorische Dynamik beurteilen	☐	
100% O_2 applizieren mit Non-Rebreather Maske	☐	
Inspektion/Bradypnoe sofort erkennen, Patient mit Ambubeutel und 100% O_2 beatmen (Thorax hebt und senkt sich)	☐	**2 PKT**
Im Verlauf Bradypnoe/resp. Verschlechterung erkennen/mit Ambubeutel beatmen	☐	
Intubation diskutieren	☐	
Intubation korrekt vorbereiten	☐	
Nicht erfolgte Beatmung mit Ambu-Beutel	☐	**-5 PKT**

Punkte /10

KREISLAUF		
Herzfrequenz/Blutdruck erheben	☐	

Pulse kontrollieren, periphere Durchblutung	☐	
Rekapillarisierungszeit	☐	
Vasovagales Manöver durchgeführt	☐	
1x peripheren venösen Zugang legen (herznah, z.B. li Ellenbeuge)	☐	
Volumen 10ml/kg	☐	
Schmalkomplex-Tachykardie erkennen: SVT richtig beschreiben (keine P Wellen, invariable Frequenz)	☐	**2 PKT**
Defi holen und Patchs ankleben	☐	
Patient mit EKG 12 Kanal monitorisieren	☐	
Adenosin: 1. Gabe (korrekte Dosierung und Verabreichung inklusive sofortigem NaCl 0.9% Bolus)	☐	**2 PKT**
Rhythmusanalyse (erfolgreiche medikamentöse Konversion?)		
Adenosin 2. Und 3. Gabe (korrekte Dosierung und Verabreichung inklusive sofortigem NaCl 0.9% Bolus)	☐	**2 PKT**
Rhythmusanalyse (erfolgreiche medikamentöse Konversion?)		
Kardioversion: Defibrillator korrekt einstellen, korrekte Energiemenge	☐	**2 PKT**

Punkte **/18**

**DISABILITY**		
GCS	☐	
Pupillen	☐	
Blutzucker kontrollieren	☐	

Punkte: **/3**

**EXPOSURE**		
Temperatur	☐	
Verletzungen	☐	

Punkte: **/2**

LEADERSHIP/KOMMUNIKATION		
**Teamleader**<br>- Klare Rollen- und Aufgabenverteilung<br>o Nicht beobachtet 0<br>o Vereinzelt beobachtet 1<br>o Immer wieder beobachtet 2<br>o Oft beobachtet 3<br>o Sehr oft beobachtet 4	☐<br>☐<br>☐<br>☐	**4 PKT**
Strukturiert ABCDE durchführen (0-4)<br>o Nicht beobachtet 0<br>o Vereinzelt beobachtet 1<br>o Immer wieder beobachtet 2<br>o Oft beobachtet 3<br>o Sehr oft beobachtet 4	☐<br>☐<br>☐<br>☐	**4 PKT**
**Teamkommunikation**<br>- Mit Namen (o.ä., z.B. Funktion) ansprechen<br>o Nicht beobachtet 0<br>o Vereinzelt beobachtet 1<br>o Immer wieder beobachtet 2<br>o Oft beobachtet 3<br>o Sehr oft beobachtet 4	☐<br>☐<br>☐<br>☐	**4 PKT**
- Speak up<br>o Nicht beobachtet 0<br>o Vereinzelt beobachtet 1<br>o Immer wieder beobachtet 2<br>o Oft beobachtet 3<br>o Sehr oft beobachtet 4	☐<br>☐<br>☐<br>☐	**4 PKT**
- Closed-loop Communication<br>o Nicht beobachtet 0<br>o Vereinzelt beobachtet 1<br>o Immer wieder beobachtet 2<br>o Oft beobachtet 3<br>o Sehr oft beobachtet 4	☐<br>☐<br>☐<br>☐	**4 PKT**
- 10 for 10 oder Stop (Re-evaluation)<br>o Nicht beobachtet 0<br>o Vereinzelt beobachtet 1<br>o Immer wieder beobachtet 2<br>o Oft beobachtet 3<br>o Sehr oft beobachtet 4	☐<br>☐<br>☐<br>☐	**4 PKT**
- Hilfe anfordern (z.B. Kardiologen)		**1 PKT**

Punkte: /25

Gesamtpunktzahl: /60
