# Supplementary material for: Avatar and distance simulation as a learning tool – virtual simulation technology as a facilitator or barrier? A questionnaire-based study on behalf of Netzwerk Kindersimulation e.V
Source: Front Pediatr. 2022 Oct 26;10:853243. doi: 10.3389/fped.2022.853243 (PMC9644191; doi:10.3389/fped.2022.853243)
Supplement: Supplementary file 4 [file Datasheet4.pdf]

NETZWERK KINDERSIMULATION

Team: **Szenario: Ertrinkungsunfall mit Rea**

**ATEMWEGE**		
Atemwege Check	☐	
Atemwege frei legen (Wasser absaugen)	☐	
HWS stabilisieren	☐	

Punkte: /3

**ATMUNG**		
Atemfrequenz checken	☐	
SaO_2% messen/Monitoring	☐	
Auskultation	☐	
Atemarbeit/respiratorische Dynamik beurteilen	☐	
100% O_2 applizieren mit Non-Rebreather Maske	☐	
Inspektion/ Atemstillstand erkennen, korrekt mit Ambubeutel und 100% O_2 beatmen (Thorax hebt und senkt sich)	☐	**2 PKT**
Intubation diskutieren	☐	
Intubation korrekt vorbereiten	☐	
Nicht erfolgte Beatmung mit Ambu-Beutel	☐	**-5 PKT**

Punkte: /9

**KREISLAUF**		
Herzfrequenz/Blutdruck erheben	☐	
Pulse kontrollieren, periphere Durchblutung	☐	
Rekapillarisierungszeit	☐	
Peripher venöser Zugang (maximal 2 Versuche)	☐	
Intraossären Zugang legen<br>- Korrekte Technik<br>- Desinfektion<br>- Lokalanästhesie<br>- Handschuhe<br>- Aspiration (Lagekontrolle)		
PEA erkennen und Ursachen (H's und T's) diskutieren	☐	**2 PKT**
REA starten, korrekter Algorithmus (5 initiale Beatmungen/15:2, nicht schockbarer Rhythmus)	☐	**2 PKT**
Defi holen und Patchs ankleben	☐	
Adrenalin korrekt vorbereiten und verabreichen (1:10 000, 0.1ml/kg)	☐	**2 PKT**
Kammerflimmern erkennen	☐	**2 PKT**
Korrekt defibrillieren (4 J/kg)	☐	**2 PKT**
Nach der Defibrillation direkte Wiederaufnahme der CPR	☐	
REA nicht sofort gestartet (<15 Sek)	☐	**- 5 PKT**

Punkte: /17

**DISABILITY**		
GCS	☐	
Pupillen	☐	
Blutzucker	☐	

Punkte: /3

**EXPOSURE**		
Temperatur	☐	
Hypothermie erkennen und behandeln<br>- Bare hugger<br>- Decke<br>- Warme Infusion<br>- Nasse Kleider ausziehen	☐	
Logroll	☐	

Punkte: /3 (35)

**LEADERSHIP/KOMMUNIKATION**		
**Teamleader**<br>- Klare Rollen- und Aufgabenverteilung<br>o Nicht beobachtet 0<br>o Vereinzelt beobachtet 1<br>o Immer wieder beobachtet 2<br>o Oft beobachtet 3<br>o Sehr oft beobachtet 4	☐<br>☐<br>☐<br>☐	**4 PKT**
Strukturiert ABCDE durchführen (0-4)<br>o Nicht beobachtet 0<br>o Vereinzelt beobachtet 1<br>o Immer wieder beobachtet 2<br>o Oft beobachtet 3<br>o Sehr oft beobachtet 4	☐<br>☐<br>☐<br>☐	**4 PKT**
**Teamkommunikation**<br>- Mit Namen (o.ä., z.B. Funktion) ansprechen<br>o Nicht beobachtet 0<br>o Vereinzelt beobachtet 1<br>o Immer wieder beobachtet 2<br>o Oft beobachtet 3<br>o Sehr oft beobachtet 4	☐<br>☐<br>☐<br>☐	**4 PKT**
- Speak up<br>o Nicht beobachtet 0<br>o Vereinzelt beobachtet 1<br>o Immer wieder beobachtet 2<br>o Oft beobachtet 3<br>o Sehr oft beobachtet 4	☐<br>☐<br>☐<br>☐	**4 PKT**
- Closed-loop Communication<br>o Nicht beobachtet 0<br>o Vereinzelt beobachtet 1<br>o Immer wieder beobachtet 2	☐<br>☐	**4 PKT**

○ Oft beobachtet 3<br>○ Sehr oft beobachtet 4	☐<br>☐	
- 10 for 10 oder Stop (Re-evaluation)<br>○ Nicht beobachtet 0<br>○ Vereinzelt beobachtet 1<br>○ Immer wieder beobachtet 2<br>○ Oft beobachtet 3<br>○ Sehr oft beobachtet 4	☐<br>☐<br>☐<br>☐	**4 PKT**
- Hilfe anfordern (z.B. Oberarzt)		**1 PKT**

Punkte: /25

Gesamtpunktzahl: /60
