# Supplementary material for: Avatar and distance simulation as a learning tool – virtual simulation technology as a facilitator or barrier? A questionnaire-based study on behalf of Netzwerk Kindersimulation e.V
Source: Front Pediatr. 2022 Oct 26;10:853243. doi: 10.3389/fped.2022.853243 (PMC9644191; doi:10.3389/fped.2022.853243)
Supplement: Supplementary file 5 [file Datasheet5.pdf]

NETZWERK KINDERSIMULATION

Team: **Szenario: Epileptischer Anfall**

**ATEMWEGE**		
Check Atemwege	☐	
Absaugen nasales Sekret	☐	

Punkte: /2

**ATMUNG**		
Atemfrequenz checken	☐	
SaO_2% messen	☐	
Auskultation	☐	
Atemarbeit/respiratorische Dynamik beurteilen	☐	
100% O_2 applizieren mit Non-Rebreather Maske	☐	
Inspektion/sofort Bradypnoe erkennen	☐	
Im Verlauf Bradypnoe/resp. Verschlechterung erkennen/mit Beatmungsbeutel beatmen	☐	
Guedel/Wendeltubus anwenden	☐	
Nicht erfolgte Beatmung mit Ambu-Beutel	☐	**-5 PKT**

Punkte: /8

KREISLAUF		
Herzfrequenz/Blutdruck erheben	☐	
Pulse kontrollieren, periphere Durchblutung	☐	
Rekapillarisierungszeit	☐	
Herzrhythmus Check	☐	
Peripheren venösen Zugang legen	☐	
Intraossären Zugang legen<br>• Korrekte Technik<br>• Desinfektion<br>• Lokalanästhesie<br>• Handschuhe<br>Aspiration (Lagekontrolle)	☐	
2. periphervenösen Zugang legen	☐	
Antibiose i.v. beginnen	☐	
Volumen 2x10ml/kg	☐	
Labor: Chemie, Gerinnung, Blutbild, BGA, Blutkultur	☐	
Analgesie, Antipyretika	☐	

Punkte: /11

DISABILITY		
GCS/ACVPU	☐	
Pupillen	☐	
Erste Benzodiazepin Gabe<br>- Midazolam im 0.2mg/kg (max 10mg)<br>- Intranasal/buccal 0.3mg/kg<br>- I.v. 0.15mg/kg (max 7.5mg)<br>- Lorazepam iv 0.1mg/kg (max 4mg)<br>- Diazepam iv 0.2 - 0.25 mg (max 10mg), rectal 0.5mg/Kg (max 20mg)	☐	**2 PKT**
Zweite Benzodiazepin Gabe	☐	**2 PKT**

Dritte Gabe Antikonvulsivum<br>- Levetiracetam<br>- Phenytoin<br>- Phenobarbital<br>- Valproat<br>(Levetiracetam 40-60 mg/kg iv (max 4.5g), Phenytoin 20mg/Kg iv (max 1.5g), Valproat 40mg/kg iv (max 3g), Phenobarbital 20mg/kg)	☐	**2 PKT**
Blutzucker	☐	
Hypoglykämie-> Bolus mit Glukose 10% 3 ml/kg	☐	**2 PKT**
Blutzucker nachkontrollieren	☐	
Hypoglykämie nicht korrigiert		**-5 PKT**
Benzodiazepin nicht verabreicht		**-5 PKT**

Punkte: /12

**EXPOSURE**		
Temperatur	☐	
Patient komplett entkleiden	☐	

Punkte: /2 (35)

**LEADERSHIP/KOMMUNIKATION**		
**Teamleader**<br>- Klare Rollen- und Aufgabenverteilung<br>○ Nicht beobachtet 0<br>○ Vereinzelt beobachtet 1<br>○ Immer wieder beobachtet 2<br>○ Oft beobachtet 3<br>○ Sehr oft beobachtet 4	☐<br>☐<br>☐<br>☐	**4 PKT**
Strukturiert ABCDE durchführen (0-4)<br>○ Nicht beobachtet 0<br>○ Vereinzelt beobachtet 1<br>○ Immer wieder beobachtet 2<br>○ Oft beobachtet 3<br>○ Sehr oft beobachtet 4	☐<br>☐<br>☐<br>☐	**4 PKT**
**Teamkommunikation**		**4 PKT**

- Mit Namen (o.ä., z.B. Funktion) ansprechen<br>o Nicht beobachtet 0<br>o Vereinzelt beobachtet 1<br>o Immer wieder beobachtet 2<br>o Oft beobachtet 3<br>o Sehr oft beobachtet 4	□<br>□<br>□<br>□	
- Speak up<br>o Nicht beobachtet 0<br>o Vereinzelt beobachtet 1<br>o Immer wieder beobachtet 2<br>o Oft beobachtet 3<br>o Sehr oft beobachtet 4	□<br>□<br>□<br>□	**4 PKT**
- Closed-loop Communication<br>o Nicht beobachtet 0<br>o Vereinzelt beobachtet 1<br>o Immer wieder beobachtet 2<br>o Oft beobachtet 3<br>o Sehr oft beobachtet 4	□<br>□<br>□<br>□	**4 PKT**
- 10 for 10 oder Stop (Re-evaluation)<br>o Nicht beobachtet 0<br>o Vereinzelt beobachtet 1<br>o Immer wieder beobachtet 2<br>o Oft beobachtet 3<br>o Sehr oft beobachtet 4	□<br>□<br>□<br>□	**4 PKT**
- Hilfe anfordern (z.B. Neurologe)		**1 PKT**

Punkte: /25

Gesamtpunktzahl: /60
